# Bispectral index detects intraoperative cerebral ischaemia during balloon assisted cerebral aneurysm coiling

**DOI:** 10.12688/f1000research.2-225.v2

**Published:** 2014-02-13

**Authors:** Zoe Harclerode, John Andrzejowski, Stuart Coley, Richard Dyde

**Affiliations:** 1 Department of Anaesthesia, Royal Hallamshire Hospital, Sheffield, S10 2JF, UK; 2 Department of Radiology, Royal Hallamshire Hospital, Sheffield, S10 2JF, UK

## Abstract

Bispectral index (BIS) is a monitoring modality designed and used for monitoring depth of anaesthesia. We wish to report a case where BIS monitoring may have alerted us to a potential adverse neurological event during angiographic coiling of a cerebral aneurysm.

## Case

Bispectral index (BIS) is a monitoring modality designed and used for monitoring depth of anaesthesia. However, it may also have other advantages in detecting intracranial haemodynamic events ^1– 3^. We wish to report a case where BIS monitoring may have alerted us to a potential adverse neurological event during angiographic coiling of a cerebral aneurysm.

**Figure 1.**
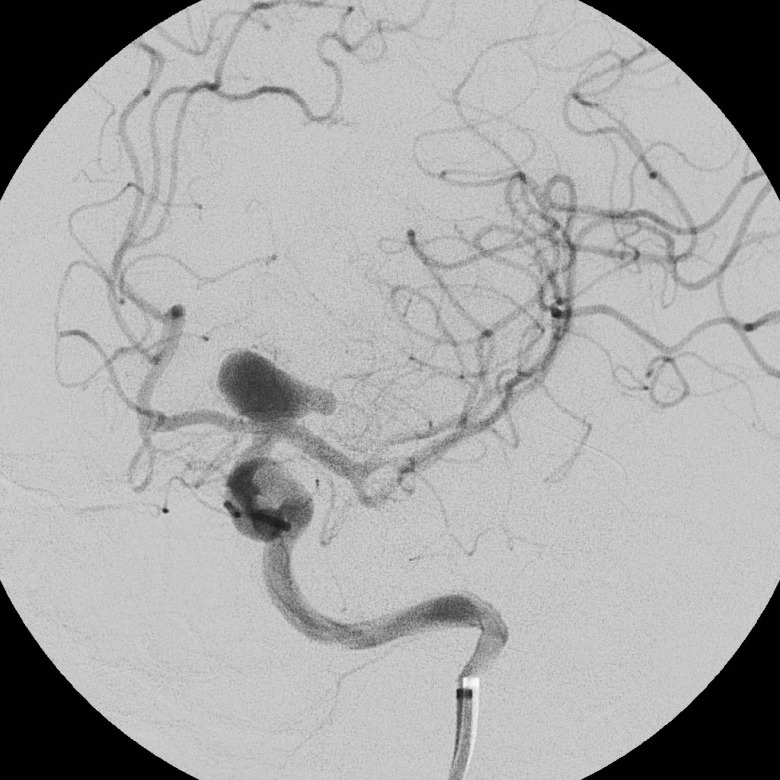
Cerebral angiography demonstrated a wide necked aneurysm at the termination of the ICA.

A 56 year old, right handed male was listed for coiling of an unruptured intracranial aneurysm. The aneurysm was discovered as an incidental finding on a CT scan of the head performed for the investigation of a previous episode of confusion. Cerebral angiography demonstrated an approximately 12mm wide necked aneurysm at the termination of the intracranial segment of the left internal carotid artery (ICA) ( Figure 1). The patient’s past medical history included hypertension and heavy smoking and he had a BMI of 32.

A small premedication dose of 2mg intravenous midazolam was given whilst an arterial line was inserted to allow continuous invasive intra-arterial blood pressure monitoring. Standard AAGBI monitoring was supplemented with BIS (Aspect Medical Systems, Newton MA, USA) that was positioned over the forehead and left temporal area before induction. An infusion of TCI (target controlled infusion) remifentanil was then commenced with an effect site (Cet) of 2ng/ml increasing to 4ng/ml before induction, which consisted of 140mg propofol, with rocuronium 60mg given to facilitate tracheal intubation.

Anaesthesia was maintained with sevoflurane (end tidal concentration 1.2–1.4) and TCI remifentanil continued at Cet of 4ng/ml, targeting a BIS range of 40–55. A metaraminol infusion was used to maintain a systolic blood pressure between 110–130mmHg. End tidal CO _2_ was maintained in the range of 4.5–5.1kPa.

Coiling was performed with vascular access obtained via a right femoral arterial puncture. Using a guide catheter placed in the extra-cranial segment of the left ICA, a balloon catheter was navigated into the intracranial circulation. The inflatable/deflatable 4mm balloon was positioned across the neck of the aneurysm and during inflation there was simultaneous occlusion of the neck of the aneurysm and the proximal segment of the left middle cerebral artery (MCA). The balloon was intermittently inflated across the neck of the aneurysm to prevent coil prolapse during placement of 11 platinum microcoils into the aneurysm. Each inflation lasted in the order of 30–120 seconds. An intravenous bolus of 5000 units of Heparin was given to minimize the risk of thromboembolism associated with the endovascular devices.

**Figure 2.**
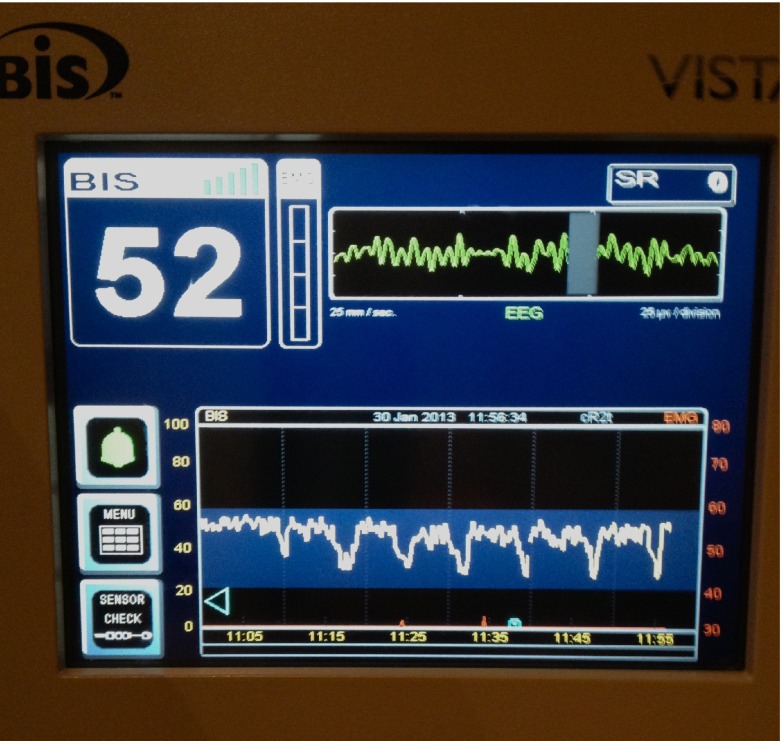
Photograph demonstrating repeated falls in BIS value with each balloon inflation, with rapid recovery to pre-inflation value upon deflation.

The photograph of the intraoperative BIS trend ( Figure 2) shows that each time the balloon was inflated, after an initial 30–40 second delay (the monitor was set to a 30 second smoothing interval), the BIS value fell to a value of approximately 25 over a 60 second period. Upon balloon deflation, BIS seemed to return more rapidly to its pre-inflation value. The red right hand scale in Figure 2 shows that there was no EMG interference that could have contributed to these changes, nor had there been any coincidental haemodynamic variation. No muscle relaxants or drugs other than those outlined above, had been administered. The anaesthetic team alerted the neuro-radiologists to these changes. They were able to limit the duration of subsequent balloon inflations resulting in demonstrably shorter subsequent BIS falls as seen in Figure 2.

Post procedure, the patient was woken up and on return to the ward had a GCS of 15 with no neurological deficit. He was discharged home after 48 hours and has recovered well at home with no problems.

## Discussion

This case illustrates a sudden, reversible and repeated decrease in intraoperative Bispectral Index (BIS) values during a balloon coiling of an intracerebral aneurysm. One possible explanation is that the monitor detected repeated periods of cerebral hypoperfusion. An abrupt decrease in BIS values not associated with changes in anaesthetic technique or haemodynamics may alert the anaesthetist to such an event. In this case there was no change in blood pressure, there had been no drugs administered such as muscle relaxants and there was no recorded EMG interference. Early communication with the surgeon or interventionist in such cases could potentially avoid procedure related neurological injury.

There have been previous case reports of sudden decreases in BIS value during other neurosurgical and neuro-radiological interventions. These include rupture of cerebral aneurysms during coiling ^1^, embolization of arterio-venous malformations ^2^ and intraventricular haemorrhage during third ventriculoscopy ^3^. Rapid increases in intracranial pressure and vasospasm have been postulated as possible causes for the decrease in BIS values.

The aneurysm in this case was on the ICA at the termination of the vessel. The angiographic balloon was placed via the ICA into the proximal MCA. Balloon inflation with simultaneous occlusion of the distal ICA and proximal middle cerebral arteries is more hazardous than the same procedure confined to the ICA as complete occlusion of the carotid tip prevents collateral flow from the contralateral circulation to the MCA (via the circle of Willis). Repeated balloon inflation within a vessel may lead to platelet aggregation or vessel wall injury with subsequent neurological injury. However, in this case the repeated acute change in the BIS values with rapid return to baseline values, suggests a haemodynamic, rather than a thromboembolic, insult to the distal circulation.

BIS has a limited ability to detect ischaemia since, similarly to Near Infra Red Spectroscopy (NIRS), in its usual montage it only monitors a *frontal lobe*. It cannot be compared to a traditional 16 channel EEG that monitors the whole brain, and should not be considered as a standard of care for detecting cerebral ischaemia.

In this patient, the BIS sensor was positioned to primarily monitor the cerebral hemisphere undergoing intervention. BIS has not been developed for the purpose of detecting cerebral ischaemia, nor is it guaranteed to do so. Studies investigating the effects of cerebral ischaemia (such as during carotid endarterectomy) on BIS have been contradictory with some demonstrating a correlation with ischaemia whilst others shown one ^4, 5^. This apparent inconsistency is at least partially explained by distal collateral circulation being able to compensate for some causes of more proximal cerebral ischaemia. Subtle ischaemia is also unlikely to be picked up using a unilateral BIS montage. The advent of a bilateral BIS sensor for general anaesthesia ^6^ may shed more light on these discrepancies and allow more subtle changes to be detected since they could potentially result in left to right hemispheric BIS differences.

Not all balloon assisted neuro-radiological procedures will result in potential ischaemia, however those involving vascular territories with poor collateral circulation might benefit from the use of BIS (ideally in a bilateral array) as a tool to detect early and potentially avoidable adverse neurological events.

## Consent

Written informed consent for publication of their clinical details and clinical images was obtained from the patient.
