# Supplementary material for: Bispectral index detects intraoperative cerebral ischaemia during balloon assisted cerebral aneurysm coiling
Source: F1000Res. 2014 Feb 13;2:225. Originally published 2013 Oct 28. [Version 2] doi: 10.12688/f1000research.2-225.v2 (PMC3938177; doi:10.12688/f1000research.2-225.v2)
Supplement: Peer review history

# Peer review: Referee response for version 2

**DOI:** 10.5256/f1000research.3808.r5230

**Published:** 2014-06-25

**Authors:** Mark Coburn

**Affiliations:** 1 Department of Anesthesiology, University Hospital RWTH Aachen, Aachen, Germany

I approve of the revisions the authors have made.

I have read this submission. I believe that I have an appropriate level of expertise to confirm that it is of an acceptable scientific standard.

# Peer review: Referee response for version 2

**DOI:** 10.5256/f1000research.3808.r3669

**Published:** 2014-02-19

**Authors:** Luzius A Steiner

**Affiliations:** 1 Department of Anaesthesia, University Hospital Basel, Basel, Switzerland

Thank you for revising your manusript. In this revised form I do not have any further reservations.

I have read this submission. I believe that I have an appropriate level of expertise to confirm that it is of an acceptable scientific standard.

# Peer review: Referee response for version 1

**DOI:** 10.5256/f1000research.2735.r2943

**Published:** 2014-01-30

**Authors:** Mark Coburn

**Affiliations:** 1 Department of Anesthesiology, University Hospital RWTH Aachen, Aachen, Germany

The conclusions which the authors draw from this case report are far too strong and need to be adapted.

I have read this submission. I believe that I have an appropriate level of expertise to confirm that it is of an acceptable scientific standard, however I have significant reservations, as outlined above.

# Peer review: Referee response for version 1

**DOI:** 10.5256/f1000research.2735.r2216

**Published:** 2013-12-27

**Authors:** Luzius A Steiner

**Affiliations:** 1 Department of Anaesthesia, University Hospital Basel, Basel, Switzerland

This is an interesting case report. However, the discussion is somewhat superficial. I have two comments:

1. Please make it clear that the sensitivity to detect cerebral ischemia by EEG is not perfect and that the BIS monitor has not been developed for this purpose. Please explain why you think that an ischemic event would lead to a decrease in the BIS value from approximately 50 to 25 in your case (but not in all cases e.g. your references).
2. What is the temporal relationship between the balloon inflation and the BIS value. You state that it "fell to approximately 25 over a 60 second period". Theoretically, I would expect some delay before the BIS reacts (already because it samples over 15 or 30 seconds to calculate the BIS value; which sampling interval did you use?). Is there an alternative explanation you could offer (e.g. did you check the EMG value)? Did you administer neuromuscular blockers or other drugs prior to balloon inflation, is it possible that you recorded an artifact of some type? Please expand your case description and the discussion.

I have read this submission. I believe that I have an appropriate level of expertise to confirm that it is of an acceptable scientific standard, however I have significant reservations, as outlined above.

# Peer review: Referee response for version 1

**DOI:** 10.5256/f1000research.2735.r2214

**Published:** 2013-11-05

**Authors:** Ehab Farag

**Affiliations:** 1 Department of General Anesthesiology and Outcomes Research, The Cleveland Clinic Foundation, Cleveland, OH, USA

The authors should mention that BIS has a very limited ability to detect ischemia as it only monitors the frontal lobe. Therefore it cannot be compared to traditional 16 channels EEG or NIRS to detect cerebral ischemia. This case should not be used as standard for care for using BIS as a monitor for cerebral ischemia.

I have read this submission. I believe that I have an appropriate level of expertise to confirm that it is of an acceptable scientific standard, however I have significant reservations, as outlined above.

# Response

**Published:** 2013-11-13

**Authors:** John Andrzejowski

**Affiliations:** Department of Anaesthesia, Royal Hallamshire Hospital, UK

I agree with Dr Farag's comments. I would be happy to change the discussion to read: 

*BIS has a limited ability to detect ischemia since, similarly to Near Infra Red Spectroscopy (NIRS), in its usual montage it only monitors a frontal lobe. It cannot be compared to a traditional 16 channel EEG that monitors the whole brain, and should not be considered as a standard of care for detecting cerebral ischaemia.*

In this patient, the BIS sensor was positioned to primarily monitor the cerebral hemisphere undergoing intervention.

Studies investigating the effects of cerebral ischaemia (such as during carotid endarterectomy) on BIS have been contradictory with some demonstrating a correlation with ischaemia whilst others show none ^4, 5^. The advent of a bilateral BIS sensor for general anaesthesia ^6^ may shed more light on these discrepancies and allow more subtle ischaemic changes (possibly resulting in left to right BIS differences) to be detected.
